# Travellers returning ill from the tropics – a descriptive retrospective study

**DOI:** 10.1186/s40794-016-0021-1

**Published:** 2016-03-25

**Authors:** Petra Zimmermann, Konrad Mühlethaler, Hansjakob Furrer, Cornelia Staehelin

**Affiliations:** 1 Infectious Diseases Unit, University Children’s Hospital Berne, Freiburgstrasse 10, Berne, 3010 Switzerland; 2 Infectious Diseases Unit, The Royal Children’s Hospital Melbourne, 50 Flemington Road, Parkville, Australia; 3 Institute for Infectious Diseases, University of Bern, Friedbühlstrasse 51, Berne, 3001 Switzerland; 4 Department of Infectious Diseases, Bern University Hospital, University of Bern, Freiburgstrasse 10, Berne, 3010 Switzerland

**Keywords:** Returning travellers, Tropics, Pre-travel advice, International travel

## Abstract

International travel continues to increase in frequency. Health care providers need a wide understanding of the spectrum of travel related diseases and their management. This retrospective study analyses the demographic and clinical data of 360 travellers returning from the tropics presenting to an outpatient clinic at a tertiary hospital between 2003 - 2007. The aim of this study was to analyse the frequency of presenting symptoms and diseases in ill returning travellers and to correlate them to the areas visited and the duration and purpose of travel. The main symptoms during travel were diarrhoea (*n* = 200, 56 %) and fever (*n* = 124, 34 %). Travellers not visiting friends and relatives but with close contact to the local population were at more than two-fold increased risk of diarrhoea (Odds Ratio [OR] 2.5; 95 % confidence interval [CI] 1.1-6.0, *p* = 0.03) and fever (OR 2.4; 95 % CI 1.1-5.3; *p* = 0.02) compared to tourist travellers. Travellers visiting friends and relatives (VFR) were not at increased risk for diarrhoea (OR 0.6; 95 % CI 0.3-1.3; *p* = 0.17), or fever (OR 1.5; 95 % CI 0.7-3.4; *p* = 0.28). Thirty-two percent of all travellers (*n* = 115) were diagnosed with a specific pathogen. Malaria (6 %), giardiasis (6 %) and amebiasis (4 %) were the most frequently detected pathogens. The odds of malaria as a cause of the presenting illness was lower among travellers reporting pre-travel advice. Specific antimicrobial treatment was required in around one third of the patients.

## Introduction

International tourist arrivals grew by 4.4 % in 2015 and reached a total of 1,184 million in 2015 [1]. In Switzerland alone, 1.5 million travel episodes to destinations outside of Europe were recorded in 2014 [2]. There is a relative decline in people travelling for tourism, while the proportion of travellers visiting friends and relatives is increasing [3]. Approximately 8 % of travellers require medical care during or after their travel [4]. Diarrhoea and fever are the most frequent health problems affecting travellers to developing countries [5, 6]. Large surveillance studies report Asia and Sub-Saharan Africa to be the most common regions where illnesses are acquired [7]. Pre-travel advice is sought in varying degrees in international travellers: 40 – 56 % in two large series [8, 9]. We aimed to understand the specific issues of returning Swiss travellers presenting to the Department of Infectious Diseases at Bern University Hospital, Switzerland. Therefore, the frequency of presenting symptoms and diseases in ill returning travellers were analysed and correlated to the areas visited and to the duration and purpose of travel.

## Methods

The study was designed as a single centre descriptive retrospective data analysis of travellers returning ill from the tropics with a presumed travel-associated condition seeking medical care at the outpatient clinic of the Department of Infectious Diseases (ID) at the University Hospital of Berne, Switzerland between 2003 – 2007. The Infectious Disease department of University Hospital of Berne is a tertiary care institution. Most patients are referred by their family physicians for a second opinion. There are only two other physicians in the city with a specialisation in tropical diseases, therefore, for patients returning ill from the tropics, the ID department also has the role as a primary care institution.

The protocol of the study was reviewed and approved by the Department of Teaching and Research of the University Hospital of Berne. In agreement with local legal requirements at the start of the study, the protocol was judged not to be subject to approval by a constituted Competent Ethics Committee.

A predefined questionnaire was used to extract the data from the patient’s files. Data were analysed using the statistical package (StataSE11, StataCorp, College Station, TX, USA). Descriptive statistical analysis was performed and risk factors for different outcomes were evaluated using chi-square statistics for categorical variables, and parametric and non-parametric tests for numeric variables. We performed univariable logistic regression for risk factors associated with illness during and after travel.

Stool samples were analysed as follows: a routine commercial fecal concentrator system was used to provide clean sediments for microscopic examination for the identification of helminth eggs and larvae, protozoan cysts, coccidian oocysts, and microsporidian spores. *Salmonella* spp., *Shigella* spp. and *Campylobacter* spp. were routinely isolated by culture. For *Giardia lamblia (intestinalis)* and *Cryptosporidium* spp. we used solid phase immunoassays which detect Giardia specific antigen (GSA 65) respectively Cryptosporidium specific antigen (CSA). In order to distinguish between the pathogenic *Entamoeba histolytica* and the apathogenic *Entamoeba dispar* (both are microscopically indistinguishable) an enzyme immunoassay for the rapid detection of the adhesion of *E. histolytica* was used. For the detection of *Clostridium difficile* toxin and the various toxins of pathogenic *Echerichia coli* (EPEC, EHEC, ETEC, EIEC and EAEC) immunoassays and/or polymerase chain reaction (PCR) were applied on demand.

Travellers were grouped into five geographic areas according to their travel destinations: Latin America (LAM), North Africa/Middle East (NAME), Sub-Saharan Africa (SSA), Southeast and South Asia (SESA) and travellers who had visited more than one area. Diarrhoea was defined as three or more unformed bowel movements a day. Fever was defined as a body temperature above 38.5 °C when measured at our clinic. Fever during travel was self-reported and not documented. Travellers were classified as visiting friends and relatives (VFR) according to the following criteria: a traveller whose primary purpose of travel is to visit friends or relatives where there is a gradient of epidemiological risk between home and destination [10]. Travellers were classified as having close contact to the local population when they were either living with locals or working within local communities (e.g. missionary or volunteer aid worker). Travellers were further categorised as tourists or as travellers for the purpose of work or study.

## Results

The population included 360 travellers aged 11 to 82 years (mean: 35 years); 69 % were female. The majority of patients in this study (254, 71 %) came to our clinic for their first consultation after travelling while the remaining 106 patients were referred from an external doctor. One-hundred-twenty-eight (36 %) travellers returned ill from SESA, 125 (35 %) from SSA, followed by 83 (23 %) from LAM. Six travellers (2 %) travelled to more than one of these regions. Travel purpose was mainly for tourism (*n* = 210, 58 %), 31 (9 %) were VFR and 30 (8 %) had close contact to the local population. The median duration of travel was 30 days (range 5 - 540). The characteristics of ill returned travellers according to the region visited are summarized in Table 1.

**Table 1:** Characteristics of ill returned travellers according to the region visited

	All regions (% of total travellers)	Latin America (LAM)	North Africa Middle East (NAME)	Sub-Saharan Africa (SSA)	South-east and South Asia (SESA)	>1 area
Number of travellers	360	83 (23 %)	18 (5 %)	125 (35 %)	128 (36 %)	6 (2 %)
Female sex	212 (59 %)	51 (61 %)	13 (72 %)	75 (60 %)	68 (53 %)	5 (83 %)
Age Group (years)						
<30	155 (43 %)	44 (53 %)	8 (44 %)	57 (46 %)	42 (33 %)	4 (67 %)
31 – 64	197 (55 %)	38 (45 %)	10 (56 %)	64 (51 %)	83 (65 %)	2 (33 %)
>64	8 (2 %)	1 (1 %)	-	4 (3 %)	3 (2 %)	-
Duration of travel (days)						
median in d	30	36	14	30	28	91
range in d	(5 – 540)	(6 – 420)	(7 – 60)	(5 – 540)	(7 – 360)	(12 – 191)
≤14	181 (50 %)	34 (41 %)	14 (78 %)	65 (52 %)	67 (52 %)	1 (17 %)
31 – 90	78 (22 %)	24 (29 %)	1 (5 %)	21 (17 %)	31 (24 %)	1 (17 %)
≥91	79 (22 %)	22 (26 %)	-	34 (27 %)	20 (16 %)	3 (50 %)
Unknown	22 (6 %)	3 (4 %)	3 (17 %)	5 (4 %)	10 (8 %)	1 (17 %)
Travel Style						
Tourism	210 (58 %)	59 (71 %)	13 (72 %)	48 (38 %)	86 (67 %)	4 (67 %)
Close contact to locals	30 (8 %)	4 (5 %)	-	19 (15 %)	6 (5 %)	1 (17 %)
Work or study	39 (11 %)	8 (10 %)	1 (6 %)	24 (19 %)	6 (5 %)	-
VFR	31 (9 %)	4 (5 %)	2 (11 %)	15 (12 %)	9 (7 %)	1 (17 %)
Unknown	50 (14 %)	8 (10 %)	2 (11 %)	19 (15 %)	21 (16 %)	-
Pre-travel health advice	242 (67 %)	60 (72 %)	2 (11 %)	92 (74 %)	82 (64 %)	6 (100 %)
Diarrhoea during journey	200 (56 %)	49 (59 %)	14 (78 %)	79 (63 %)	54 (42 %)	4 (67 %)
Self-reported fever during journey	124 (34 %)	24 (29 %)	5 (28 %)	58 (46 %)	36 (28 %)	1 (17 %)
Presentation because of gastrointestinal complaints	182 (51 %)	51 (61 %)	12 (67 %)	51 (41 %)	64 (50 %)	4 (67 %)
Unspecific gastrointestinal disorder	129 (36 %)	35 (42 %)	12 (67 %)	32 (26 %)	47 (37 %)	3 (50 %)
Giardiasis	21 (6 %)	4 (5 %)	-	9 (7 %)	8 (6 %)	-
Amebiasis	14 (4 %)	5 (6 %)	-	4 (3 %)	4 (3 %)	1 (17 %)
Salmonellosis (non-typhoidal)	5 (1 %)	3 (4 %)	-	1 (<1 %)	1 (<1 %)	-
Campylobacteriosis	2 (<1 %)	-	-	1 (<1 %)	1 (<1 %)	-
Shigellosis	4 (1 %)	-	-	3 (2 %)	1 (<1 %)	-
Cyclosporiasis cayetanensis	3 (<1 %)	2 (2 %)	-	-	1 (<1 %)	-
Typhoid fever	2 (<1 %)	-	-	1 (<1 %)	1 (<1 %)	-
Helminthiasis	2 (<1 %)	2 (2 %)	-	-	-	-
Presentation because of fever	98 (27 %)	17 (20 %)	2 (11 %)	42 (34 %)	36 (28 %)	1 (17 %)
Upper respiratory tract infection	44 (12 %)	9 (11 %)	1 (6 %)	13 (10 %)	20 (16 %)	1 (17 %)
Fever with no clinical focus	24 (7 %)	4 (5 %)	-	15 (12 %)	5 (4 %)	-
Malaria	23 (6 %)	2 (2 %)	-	14 (11 %)	7 (5 %)	-
Dengue fever	6 (2 %)	1 (1 %)	1 (6 %)	-	4 (3 %)	-
Tuberculosis	1 (<1 %)	1 (1 %)	-	-	-	-
Medical assistance during journey	80 (22 %)	21 (25 %)	1 (6 %)	39 (31 %)	17 (13 %)	2 (33 %)

### Symptoms and medical assistance during travel

Two hundred travellers (56 %) suffered from diarrhoea during their trip. Self-reported fever was the second most common complaint during travels (*n* = 124, 34 %). Compared to tourists, travellers with close contact to the population had a more than two-fold (OR 2.5; 95 % CI 1.1-6.0, *p* = 0.03) increased odds of suffering from diarrhoea and a 2.4-fold increased risk of having fever (95 % CI 1.1-5.3; *p* =0.02) (Table 2). VFRs were not at higher risk of suffering from fever or diarrhoea. Travel of longer than three months was associated with a significantly increased rate of diarrhoea, while the occurrence of fever increased with the length of stay up to three months, but did not increase thereafter (Table 2).

**Table 2:** Proportion and odds ratio of diarrhoea or fever among all illness types according to travel style and duration of travel

	Diarrhoea		Fever	
Total	200 (56 %)	Odds ratio compared to tourists	124 (34 %)	Odds ratio compared to tourists
Travel style				
Tourism (*n* = 210, 58 %)	109 (52 %)	1	61 (29 %)	1
Close contact to locals (*n* = 30, 8 %)	22 (73 %)	2.5 (95 % CI 1.1-6.0; *p* = 0.03)	15 (50 %)	2.4 (95 % CI 1.1-5.3; *p* = 0.02)
Work or study (*n* = 39, 11 %)	26 (67 %)	1.9 (95 % CI 0.9-3.8; *p* = 0.09)	18 (46 %)	2.1 (95 % CI 1.0-4.1; *p* = 0.04)
VFR (*n* = 31, 9 %)	12 (39 %)	0.6 (95 % CI 0.3-1.3; *p* = 0.17)	12 (39 %)	1.5 (95 % CI 0.7-3.4; *p* = 0.28)
Unknown (*n* = 50, 14 %)	31 (62 %)	1.5 (95 % CI 0.8-2.8; *p* = 0.20)	18 (36 %)	1.4 (95 % CI 0.7-2.6; *p* = 0.34)
Duration of travel (in days)		Odds ratio compared to ≤ 14 days		Odds ratio compared to ≤ 14 days
≤14 (*n* = 67, 19 %)	31 (46 %)	1	17 (25 %)	1
15 - 30 (*n* = 107, 30 %)	53 (50 %)	1.1 (95 % CI 0.6-2.1; *p* = 0.67)	29 (27 %)	1.1 (95 % CI 0.6-2.2; *p* = 0.81)
31 – 60 (*n* = 60, 17 %)	33 (55 %)	1.4 (95 % CI 0.7-2.9; *p* = 0.32)	26 (43 %)	2.3 (95 % CI 1.1-4.8; *p* = 0.03)
61 - 90 (*n* = 24, 6 %)	15 (63 %)	1.9 (95 % CI 0.7-5.0; *p* = 0.17)	13 (54 %)	3.5 (95 % CI 1.3-9.2; *p* = 0.01)
91 - 364 (*n* = 65, 18 %)	46 (71 %)	2.8 (95 % CI 1.4-5.8; *p* < 0.01)	28 (43 %)	2.2 (95 % CI 1.1-4.7; *p* = 0.03)
≥365 (*n* = 13, 4 %)	10 (77 %)	3.9 (95 % CI 1.0-15.3; *p* = 0.04)	6 (46 %)	2.5 (95 % CI 0.7-8.6; *p* = 0.12^1^)
Unknown (*n* = 24, 6 %)	12 (50 %)		5 (21 %)	

^1^Fisher’s exact

Eighty out of the 360 travellers (23 %) sought medical assistance during their trip, mainly people who had close contact to the population (*n* = 11, 16 %; *p* = 0.06). Twenty travellers (6 % of all travellers) who sought medical care abroad were hospitalized. The main reasons for hospitalisation were malaria (*n* = 7) and gastrointestinal disorders (*n* = 6). Of the patients with gastrointestinal disorders two were diagnosed with salmonellosis, one each with shigellosis, and giardiasis and in two patients no specific diagnosis was made. Other reasons for hospitalisation were dengue fever (*n* = 1), urinary tract infection (*n* = 1), infected pilonidal sinus (*n* = 1) and febrile illness with a rash (*n* = 1). In three patients the reason for hospitalisation was not documented. The hospitalization rate was highest in travellers aged 16-30 years (*n* = 14, 9 %). Travel style was not associated with reported rates of hospitalization among those who presented ill to our clinic (*p* = 0.07).

### Medical assistance and diagnosis in Switzerland

Of the 360 travellers who presented at our clinic, 182 (51 %) did this because of gastrointestinal complaints, 98 (27 %) because of fever and 49 (14 %) because of a skin problem; 15 (4 %) reported fatigue and six (2 %) micturition problems. Seven travellers (2 %) requested a check-up without any complaints. There was no difference in the frequencies of these main symptoms between sexes (*p* = 0.4) nor different age groups (*p* = 0.8). The median time between returning from travel and presentation to the clinic was 11 days (range 0-313 days, data available for 321 patients). Thirty-eight percent of all travellers (*n* = 138) presented within the first week after returning.

Two-hundred-ninety-one patients (81 %) had a blood test performed, 179 (50 %) a faecal test and 65 (18 %) a urine test. Twenty patients (6 %) received an abdominal ultrasound, 11 (3 %) an X-ray and six (2 %) a CT scan. Nine patients had a skin smear and three patients a sputum examination. Only a very small number of patients needed an invasive procedure, such as skin biopsy (*n* = 3), pleural biopsy (*n* = 1), cerebrospinal fluid analysis (*n* = 1), gastroscopy (*n* = 2) or colonoscopy (*n* = 2).

A total of 502 stool samples from 179 patients were analysed. On those samples 363 microscopic tests, 121 cultures, 293 *Giardia lamblia*-antigen tests, 24 *Cryptosporidium*-antigen tests and 1 *Clostridium difficile*-antigen test were performed. The pathogens detected are reported in Table 1. In 71 % (*n* = 129) of the patients no pathogen was identified.

Of the 98 patients presenting with fever, the majority (*n* = 44, 45 %) were diagnosed with an upper respiratory tract infection or with fever without focal clinical findings (*n* = 24, 24 %). Malaria was diagnosed in 23 patients (24 % of the febrile patients, 6 % of all patients). The diagnosis was made on basis of a positive thick smear in 14 patients, a positive antigen test in 4 patients and both tests positive in one patient. Nine patients each were infected with *Plasmodium falciparum* respectively with *Plasmodium vivax/ovale*. In five patients (22 %) malaria was already diagnosed and treated abroad and no *Plasmodium* species could be verified; 61 % (*n* = 14) of the travellers with malaria had been to SSA, 16 % of the people travelling as VFR were diagnosed with malaria, while the rate was only 5 % in the other travellers (*p* = 0.02). Six patients (6 %) with fever were diagnosed with dengue fever, and one patient, returning from LAM, was diagnosed with tuberculosis.

The majority of patients (*n* = 212 patients, 59 %) did not require any treatment, however 12 patients (3 %) had to be hospitalized for the following reasons: two with *P. vivax/ovale* infection, one with *P. falciparum* infection, two each with bacterial enteritis and respiratory tract infections, and one each with giardiasis, amebiasis, pancreatitis, and complicated skin lesions.

### Associations with pre-travel advice

The majority of our patients (*n* = 242, 67 %) had sought pre-travel advice. The rate was 67 % (*n* = 140) in tourists, 83 % (*n* = 25) in travellers with close contact to the population, 82 % (*n* = 32) in people travelling for work or study and 32 % in VFRs (*n* = 10). Travellers aged 31-64 years less often sought pre-travel advice than the other age groups (*p* = 0.02). With the exception of those travelling to NAME, where only two (11 %) had seen a doctor before travelling, destination had no influence on the percentage of people seeking pre-travel advice (Table 1). All travellers who visited more than one region sought pre-travel advice. The most frequent vaccine distributed prior to the index travel was against hepatitis A or B (263 travellers, 73 %); 148 travellers (41 %) were vaccinated against typhoid fever, mainly with the live attenuated oral vaccine, only ten received the inactivated parenteral vaccine. 126 travellers (35 %) were vaccinated against yellow fever, 83 (22 %) against rabies, 28 (7 %) against meningococci, 11 (3 %) against tick-borne encephalitis and 4 (1 %) against Japanese encephalitis. People who had sought pre-travel advice showed higher vaccination coverage than those without pre-travel advice: 78 % versus 12 % (*p* < 0.01) were vaccinated against hepatitis A, 42 % versus 21 % (*p* < 0.01) against hepatitis A and B, 52 % versus 11 % (*p* = 0.01) against typhus, 48 % versus 9 % against yellow fever (*p* < 0.01) and 32 % versus 3 % (*p* < 0.01) against rabies.

Since the exact travel routes were not documented, it was not possible to evaluate how many persons travelled to malaria-endemic areas and should have taken malaria prophylaxis following current recommendations. One third of the ill-returning travellers (*n* = 110, 31 %) took malaria prophylaxis; those who had sought pre-travel advice did this significantly more frequently than those who had not (OR 7.6 95 % CI 3.8-15.3; *p* < 0.01). Of the 125 travellers going to SSA, 79 % (*n* = 87) had taken prophylaxis (pre-travel advice: 83 %; no pre-travel advice: 33 %). Fifty-six travellers (16 %) had carried malaria stand-by medication, mostly travellers to LAM (*n* = 30) or SESA (*n* = 23); this is in line with Swiss recommendations. Again, people with pre-travel advice were more likely to carry malaria stand-by medication on their trip. Among patients presenting with a travel related illness, those diagnosed with malaria reported having sought pre-travel medical advice less frequently compared to those with conditions other than malaria (OR 0.2; 95 % CI 0.1-0.5; *p* < 0.01). Further, among patients presenting with a travel related illness, those who had required self-administered drugs or medical assistance abroad reported having had pre-travel advice less often than those who had not required drugs (OR for having had pre-travel advice 0.5; 95 % CI 0.3-0.96; *p* = 0.03) or medical assistance abroad (OR 0.1; 95 % CI 0.1-0.2; *p* < 0.01). However, among these ill returning travellers, there was no difference in use of pre-travel advice in those presenting with diarrhoea or fever (*p* > 0.05 for both).

## Discussion

This study analyses travellers returning ill from the tropics and presenting to a Swiss outpatient tertiary care clinic. The main symptoms leading to a post-travel consultation were gastrointestinal complaints and fever. People who travelled for longer durations or had close contact to the population were more likely to return with a complaint of fever and diarrhoea compared to other chief complaints, while VFRs were not at higher risk of presenting for these conditions. It has been reported before that the proportionate rates of acute diarrhoea is lower in VFR in another Swiss study with a similar population [11].

The majority of gastrointestinal and febrile disorders (69 % each) remained without detection of a specific etiological agent. Specific antimicrobial treatment was administered to one third of the travellers. Nine percent of travellers were diagnosed with a potentially life-threatening condition, of which six percent suffered from malaria. Among the seven travellers diagnosed with malaria abroad, all stated to have been adherent with chemoprophylaxis provided. Whether this was attributable to misdiagnosis, recall bias, or breakthrough malaria could not be verified retrospectively. Malaria was a more frequent diagnosis in VFRs compared to other traveller types. Similar findings have been observed in larger surveillance studies where VFRs presented with malaria up to eight times more often than regular tourists [12–14].

The destination of travel was associated with specific diseases at presentation to the clinic. Systemic febrile illness with no clinical focus and malaria occurred most often in travellers returning from SSA; upper respiratory tract infections in travellers returning from SSA and SESA. Unspecific gastrointestinal disorders occurred with equal frequency in people returning from LAM, SSA and SESA. Our results are consistent with GeoSentinel and EuroTravNet based studies where systemic febrile illness and malaria occurred more often among travellers returning from SSA and SESA and diarrheal illness was most commonly seen among those returning from SSA, LAM and SESA [4, 7, 8]. Among those reporting illnesses associated with diarrhoea or fever, there was no difference in the rate reporting pre-travel advice compared to other illnesses. As expected, those who reported pre-travel advice had significantly higher vaccination coverage and use of malaria medication, both as prophylaxis and as stand-by options, and lower rates of self-administered medication or medical assistance abroad. In the study by Schlagenhauf et al, pre-travel advice also significantly reduced the proportionate morbidity of malaria, but was associated with an increase in diarrhoea [8].

The results of our study are limited by the small numbers and because data was only collected at one centre. Further, the results are subject to bias due to the retrospective design. As usual for such type of study there is a lack of denominator data from returning healthy travellers to inform true risk associations and therefore data is only descriptive. Also there were limitations in the detection of pathogens, especially from stool samples, because culture-independent methods like immunoassays and PCRs were only performed when explicitly asked for by the physician. The strengths of the study are the focus on and description of the pragmatic approach in a clinical setting without an *a priori* research agenda.

To summarize, this study reflects the pragmatic approach to returning ill travellers in Switzerland, where health insurance is compulsory, but the per capita monthly insurance premium can be chosen individually. The lower the monthly premium, the higher are the yearly costs that the patients have to pay out of their own pocket. Frequently patients with lower monthly premiums therefore opt not to have all analyses done if no practical consequences are likely to ensue from a result. This is typically the case in returning travellers with uncomplicated (afebrile, non-bloody) diarrhea or with fever and symptoms of a viral infection of the upper respiratory tract once malaria and dengue have been ruled out. In these patients the academic drive towards finding the causative pathogen is overruled by practical considerations of the financial implications of further diagnostics that are not likely to change therapeutical management. Apart from VFR travellers as a known at-risk population for malaria, our study has also identified non-VFR travellers engaging in close contacts with the local population to be at increased risk for diarrheal illness and fever. Even though the majority of illnesses in returning travellers are gastrointestinal symptoms of mild to moderate severity and a specific etiological is often not identified, malaria remains the most frequent potentially life-threatening disease in returning ill travellers. In patients presenting to our clinic with malaria or reporting the need for treatment or hospitalisation abroad, pre-travel advice had been more frequently omitted than sought – this may suggest a protective effect of pre-travel advice for these selected travel-related problems.

## Ethics approval and consent to participate

The protocol of the study has been approved by the department of teaching and research of the University Hospital of Berne. In agreement with local legal requirements at the start of the study, the protocol was judged not to be subject to approval by a constituted Competent Ethics Committee.

## Abbreviations

CI: confidence interval
LAM: Latin America
NAME: North Africa/Middle East
SESA: Southeast and South Asia
SSA: Sub-Saharan Africa
VFR: travellers visiting friends and relatives
